# An early LH surge may jeopardize ART outcomes of patients with PCOS and its predictive models

**DOI:** 10.1530/RAF-25-0160

**Published:** 2026-03-04

**Authors:** Xiaohui Ji, Huayang Xia, Ting Liang, Haiyan Lin, Yabo Yang, Wenqing Que, Qinxue Zhang, Hui Chen, Qi Qiu, Lina Wei, Yi Li

**Affiliations:** ^1^ Center for Reproductive Medicine, Sun Yat-Sen Memorial Hospital of Sun Yat-Sen University, Guangzhou, Guangdong, China; ^2^ Guangdong Provincial Key Laboratory of Malignant Tumor Epigenetics and Gene Regulation, Sun Yat-Sen Memorial Hospital, Sun Yat-Sen University, Guangzhou, Guangdong, China; ^3^ Preventive Medicine and Medical School, Jinan University, Guangzhou, China

**Keywords:** polycystic ovary syndrome, controlled ovarian stimulation, an early LH surge, ART outcomes, predictive models

## Abstract

**Abstract:**

This study aimed to investigate whether an early LH surge during controlled ovarian stimulation (COS) affects pregnancy outcomes in patients with polycystic ovary syndrome (PCOS) undergoing assisted reproductive technology (ART) and to explore methods for identifying high-risk patients for early intervention. In a retrospective case–control analysis of 786 PCOS cycles, an early LH surge was found to significantly increase the spontaneous abortion rate and reduce the live birth rate in *in vitro* fertilization and multiple embryo transfer cycles, while also correlating with a higher preterm birth rate in frozen embryo transfer cycles. Using the training set of 786 cycles, three predictive models for LH surge were developed and validated in an independent time validation set of 76 cycles. Among them, model 2 demonstrated optimal performance, with prediction feasible as early as COS day 0 and an AUC of 0.713. The findings indicate that an early LH surge may compromise ART outcomes in specific PCOS subgroups, and the proposed predictive models could aid clinicians in identifying high-risk patients and adapting treatment strategies accordingly.

**Lay summary:**

For women with polycystic ovary syndrome (PCOS) undergoing fertility treatment, a premature hormone surge can sometimes disrupt the process. Our study investigated how this early ‘luteinizing hormone (LH) surge’ affects pregnancy success. We found that women who experienced this early surge had more eggs collected. However, for those who had a fresh embryo transfer, it led to a higher risk of miscarriage and a lower chance of having a baby. If they used frozen embryos in a later cycle, the risk of premature birth was higher. To help patients, we also created a prediction tool. This tool uses simple information collected at the start of treatment – like a woman’s body mass index and hormone levels – to identify those at high risk of this early surge. By spotting these patients early, doctors can personalize their treatment to improve their chances of a successful and healthy pregnancy.

## Introduction

A LH surge triggers the extrusion of the first polar body in oocytes, which symbolizes the end of the first meiosis (Sen 2013, Celik *et al.* 2015, Arroyo *et al.* 2020). Therefore, an appropriate LH surge is critical for the normal maturation of oocytes and subsequent embryo development (Robker *et al.* 2018). Without intervention, a premature LH surge may occur in 20–30% of controlled ovarian stimulation (COS) cycles due to multi-follicular growth. The premature LH rise may disrupt oocyte maturation, impair embryo quality, and compromise therapeutic outcomes (Kawamura *et al.* 2004, Dozortsev & Diamond 2020). To improve clinical outcomes, GnRH antagonists or agonists were introduced to prevent premature surges during COS cycles (Sengoku *et al.* 1997, Urman *et al.* 2004).

Polycystic ovary syndrome (PCOS) affects nearly 10% of women of reproductive age (Azziz *et al.* 2004, Hart *et al.* 2004, Wolf *et al.* 2018), accounting for approximately 20–30% of COS cycles (Norman *et al.* 2007, George *et al.* 2012, Sirmans & Pate 2013). Because of the rapid rise in estradiol secreted by multiple follicles of PCOS patients, the premature LH surge commonly occurs in the early phase of ovarian stimulation. In contrast, patients with ovarian insufficiency usually suffer from a premature LH surge in the late stimulation stage due to a lower number of growing follicles, which results in a slower elevation of estradiol. The GnRH antagonist protocol is often used to prevent the early rise of LH surge in patients with PCOS (Pundir *et al.* 2012, Toftager *et al.* 2016, Kadoura *et al.* 2022). However, due to slow follicular growth in PCOS patients, the addition of the GnRH antagonist is usually delayed during the COS cycle. Consequently, an unanticipated LH surge may occur prior to GnRH antagonist treatment. Nevertheless, clinicians easily overlook the early LH surge because it can be immediately suppressed by the GnRH antagonist. Another possibility is that patients with PCOS were thought to be more resistant to the surge because of their good ovarian reserve.

To date, few studies have explored the impact of an early LH surge on the assisted reproductive technology (ART) outcomes of patients with PCOS who are essentially distinct from patients with ovarian insufficiency in terms of ovarian reserve. Assuming the early LH surge is also detrimental to pregnancy outcomes, is it possible to identify high-risk patients earlier? If the early prediction is feasible, clinicians can take preventive measures to improve therapeutic outcomes.

In brief, this study sought to examine if an early LH surge jeopardizes the ART outcomes of patients with PCOS and to develop predictive models to identify high-risk patients and improve obstetrical outcomes.

## Methods

### Study design and population

This case–control study investigated infertile patients who underwent assisted reproduction treatment at Sun Yat-Sen Memorial Hospital in Guangzhou. Before initiating the project, permission from the Institutional Review Board (IRB) was acquired. This study adhered to the Strengthening the Reporting of Observational Studies in Epidemiology (STROBE) reporting guidelines.

For the preliminary selection, patients with PCOS undergoing COS for the ART treatment were recruited. PCOS diagnosis was based on the Rotterdam 2003 criteria. In addition, the women who met only one Rotterdam criteria were excluded from the study. The inclusive criteria included i) pelvic or tubal factors identified by HSG or laparoscopy, ii) male factors, iii) pregnancy failure after three intrauterine insemination (IUI) attempts, and iv) absence of oral contraceptive and gonadotropin therapy within three months. Furthermore, the following patients were excluded from the study: i) patients without PCOS, ii) non-antagonist protocols, iii) endometriosis, iv) uterine anomaly, v) genetic abnormalities, vi) immune disorders, vii) systemic diseases, and viii) missing data.

### ART procedure

Before beginning ART cycles, all patients in our clinic were required to complete a physical examination for pregnancy three months beforehand (the basal level). Patients with PCOS were then administered a flexible GnRH antagonist regimen. The injection of gonadotropin started on the second or third day of natural menstruation or progesterone withdrawal bleeding (COS day 0). During the COS cycle, patients were scheduled to see a doctor on the fifth or sixth day after the initial gonadotropin injection (COS day 5/6). The patients would then be checked every two to four days based on ultrasound results and serum levels of sex hormones. When the diameter of dominant follicles reached 12 mm, or the estradiol level exceeded 600 pg/mL, or the LH level exceeded 10 IU/L, a GnRH antagonist would be added. HCG was administrated to trigger oocyte maturation as at least two dominant follicles larger than 18 mm. Oocyte retrieval surgery was performed 34–38 h after hCG administration. The retrieved oocytes were fertilized by *in vitro* fertilization (IVF) or intracytoplasmic injection (ICSI) and then cultured to the cleavage or blastocyst stage. The embryo transfer was conducted three or five days after the ovarian surgery. The luteal support of vaginal progestins began with the oocyte pickup day and continued to the 10th gestational week if the patient became pregnant.

### An early LH surge

Following the manufacturer’s instructions, we assessed the LH level using an automated chemiluminescent immunoassay (CLIA) platform (Beckman Coulter) and an access hLH assay kit (Beckman Coulter, #33510, USA). The intra-assay and total coefficients of variation were 3.8–5.4% and 4.3–6.4%, respectively. The kit’s test limits ranged from 0.2 to 250.0 IU/L. An early LH surge is defined as the LH level on COS day 5/6 greater than 10 IU/L and 50% higher than the baseline detected on COS day 0. The patients were classified into two groups (case and control) based on whether or not they were exposed to an early LH surge. If an early LH rise were detected, a GnRH antagonist would be added to suppress the surge, and the LH level would be checked 24 h later.

### ART outcomes

The terminology of ART outcomes is based on the 2017 ICMART definitions. Briefly, clinical pregnancy was defined as the detection of one or more gestational sacs via ultrasonography. In this study, the clinical pregnancy rate was determined by dividing the number of clinical pregnancy cycles by the number of embryo transfer cycles. In addition, spontaneous abortion was defined as the spontaneous intrauterine pregnancy loss prior to 22 completed weeks of gestation age. The spontaneous abortion rate corresponds to the ratio of spontaneous abortion cycles to clinical pregnancy cycles. Preterm birth refers to a birth that takes place between 22 and 37 completed weeks of gestation age. The ratio of preterm birth cycles divided by live birth cycles was used to represent the preterm birth rate. Finally, a live birth means an infant born after 22 completed weeks of gestation age shows postnatal evidence of life. The live birth rate equals the ratio of live birth cycles to embryo transfer cycles.

### Statistical analysis

R version 4.05 (https://www.R.project.org) was used for statistical analysis. We used the Shapiro–Wilk test to determine the normality of continuous variables, and the results indicated that the variables were abnormally distributed. Continuous variables were expressed as median (interquartile range (IQR) = *P*_75_–*P*_25_), and the differences between the two groups were compared using the Mann–Whitney U test. In addition, categorical variables were expressed as frequency (percentages) and compared using the Pearson chi-squared test or Fisher’s exact probability. Then, subgroup analysis was based on several clinical factors that may influence ART outcomes, including the fresh/frozen transfer cycle, fertilization method, transferred embryo type, transferred embryo quality, and transferred embryo number. In the first part, we utilized univariate and multivariate logistic regression to analyze whether an early LH surge jeopardizes ART outcomes, including the clinical pregnancy rate, spontaneous abortion, preterm birth rate, and the live birth rate, and calculate the odds ratio (OR) and 95% CI (confidence interval).

In the second part, we further constructed the prediction model of an early LH surge. We first identify significant prognostic factors (*P* < 0.01) using univariate logistic regression. Then, these prognostic factors were further utilized to build the final model, which contributes to an early LH surge. The training set was comprised of 786 COS cycles between January 1, 2014, and April 1, 2020. Based on the risk factors, we developed three models to estimate the likelihood of an early LH surge. Furthermore, we utilized a new cohort of 76 cycles recruited between January 1, 2021, and December 31, 2021, as the time validation set to validate our prediction model. The receiver area under the curve (AUC), calibration curve, and decision curve analysis (DCA) were used to assess the efficacy of predictive models. R version 4.05 (https://www.R.project.org) was used for statistical analysis, with *P* < 0.05 (two-tailed) considered a significant difference.

## Results

### Participants’ recruitment

**Figure 1:**
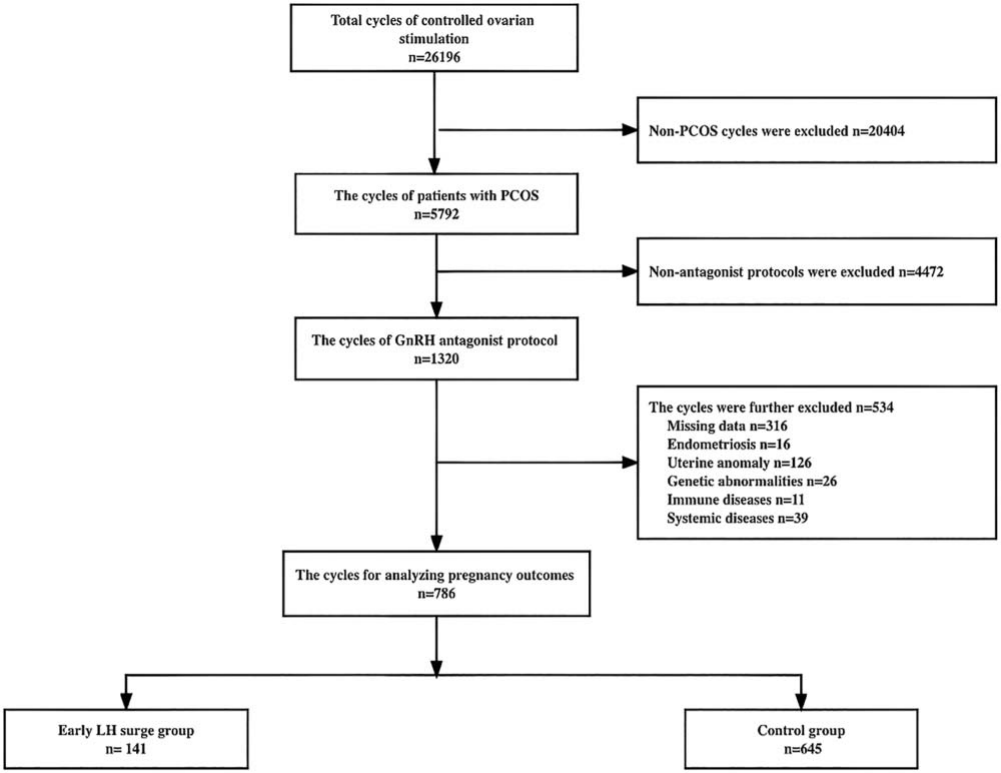
Flowchart for recruitment of patients with PCOS in the training.

Initially, a retrospective analysis of 26,196 COS cycles was performed between January 1, 2014, and April 1, 2020. The study excluded 20,404 non-PCOS cycles and 4,472 non-antagonist cycles. Furthermore, 534 patients who did not meet inclusive criteria were excluded. In the end, 786 cycles were enrolled in the training set, with 141 cycles (93 fresh transfer cycles + 48 frozen embryo cycles) in the case group and 645 cycles (299 fresh transfer cycles + 346 frozen embryo cycles) in the control group. The recruiting flowchart of training set patients is presented in Fig. 1. As for the patient recruitment of time validation set, data were collected from 2,735 COS cycles between January 1, 2021, and December 31, 2021. In the beginning, 2,463 non-PCOS cycles and 132 non-antagonist cycles were excluded from the study. In addition, 64 cycles were excluded based on the exclusive criteria. Finally, 76 cycles were enrolled in the time validation set, with 13 in the case group and 63 in the control group. The flowchart of patients’ recruitment is shown in Fig. 3.

### Characteristics of training set patients

The LH surge group had lower basal BMI, follicle-stimulating hormone (FSH), and thyroid stimulating hormone (TSH) levels but higher PRL and AMH levels than the control group (Table 1). In addition, the LH surge group had higher levels of LH, estradiol (E_2_), and progesterone on COS day 0, COS day 5/6, and hCG day. The patients with an early LH surge harvested more eggs (median 19 (15.23) vs median 14 (10.19), *P* < 0.001) but similar blastocysts (median 2 (0.5) vs median 2 (0.5), *P* = 0.617). Furthermore, the ovarian hyperstimulation rates were comparable between two groups (2.84% vs 1.71).

**Table 1:** Characteristics of training set patients.* P *values in bold indicate statistical significance (*P*<0.05).

Variables	LH surge	Control	*Z*/*χ*^2^	*P*
Age (years)	30 (27.5, 32)	30 (27, 33)	−0.22	0.829
BMI (kg/m^2^)	21.50 (18.80, 24.20)	22.30 (20.00, 24.80)	−2.28	**0.023**
Basal levels
LH (IU/L)	7.17 (5.30, 10.57)	7.07 (4.61, 10.88)	0.84	0.4
FSH (IU/L)	6.45 (5.47, 7.63)	7.04 (5.87, 8.19)	−3.19	**0.001**
E_2_ (ng/L)	43.00 (33.93, 61.00)	40.40 (28.00, 56.00)	1.66	0.096
Progesterone (μg/L)	0.51 (0.41, 1.34)	0.60 (0.37, 1.11)	−0.07	0.943
Testosterone (nmol/L)	1.97 (1.34, 2.38)	1.80 (1.31, 2.45)	0.42	0.678
PRL (μg/L)	13.26 (10.29, 18.30)	11.75 (8.68, 16.60)	2.2	**0.028**
AMH (ng/mL)	11.09 (7.69, 15.40)	8.87 (5.74, 13.36)	2.65	**0.008**
AFC	32 (24, 40)	30 (24, 36)	1.9	0.058
TSH (mU/L)	1.72 (1.06, 2.10)	1.88 (1.35, 2.44)	−2.25	**0.024**
Fasting glucose (mmol/L)	5.00 (4.80, 5.30)	5.10 (4.80, 5.30)	−0.88	0.381
Fasting insulin (mU/L)	10.49 (6.34, 15.39)	10.84 (7.59, 14.18)	−0.81	0.419
HOMA index	2.44 (1.39, 3.30)	2.41 (1.68, 3.28)	−0.71	0.478
COS day 0 levels
LH (IU/L)	6.97 (5.76, 9.15)	6.18 (4.56, 8.48)	3.08	**0.002**
FSH (IU/L)	6.48 (5.69, 7.54)	7.19 (6.15, 8.40)	−3.7	**<0.001**
E_2_ (ng/L)	45.50 (35.50, 61.50)	38.00 (2.00, 53.00)	3.65	**<0.001**
Progesterone (μg/L)	0.68 (0.46, 1.10)	0.59 (0.38, 0.92)	2.23	**0.026**
COS day 5/6 levels
LH (IU/L)	16.77 (12.78, 21.54)	3.52 (2.24, 5.06)	15.18	**<0.001**
FSH (IU/L)	13.04 (10.55, 15.31)	10.80 (8.80, 13.21)	4.95	**<0.001**
E_2_ (ng/L)	1,813.5 (1,138, 2,539)	554.5 (216.5, 972.5)	10.48	**<0.001**
Progesterone (μg/L)	1.20 (0.77, 1.53)	0.57 (0.38, 0.85)	9.4	**<0.001**
HCG day levels
LH (IU/L)	3.61 (1.95, 6.20)	2.82 (1.72, 4.60)	2.94	**0.003**
FSH (IU/L)	10.42 (9.39, 13.85)	11.71 (9.25, 15.09)	−1.88	0.06
E_2_ (ng/L)	4,839 (4,086, 4,901)	3,210 (2,005, 4,800)	6.91	**<0.001**
Progesterone (μg/L)	1.40 (1.05, 1.95)	0.93 (0.67, 1.31)	7.04	**<0.001**
Gonadotropin dose (IU)	1,050 (900, 1,293.75)	1,350 (1,093.75, 1,800)	−6.80	**<0.001**
Gonadotropin days	8 (7.9)	10 (8.11)	−6.97	**<0.001**
Retrieved eggs, *n*	19 (15.23)	14 (10.19)	5.6	**<0.001**
2 PN zygotes, *n*	11 (7.15)	8 (5.12)	4.29	**<0.001**
2 PN zygote rate (%)	62.50 (47.06, 75.00)	61.90 (46.15, 75.00)	0.02	0.988
Usable embryos, *n*	6 (4.9)	4 (2.7)	4.79	**<0.001**
Usable embryo rate (%)	61.54 (38.10, 81.82)	56.25 (38.89, 77.78)	0.83	0.405
Good embryos, *n*	3 (1.6)	3 (1.5)	2.02	**0.043**
Good embryo rate (%)	30.43 (13.04, 52.38)	31.25 (16.67, 50.00)	−0.05	0.957
Blastocysts, *n*	2 (0.5)	2 (0.5)	−0.5	0.617
Blastocyst formation rate (%)	50.00 (0.00, 75.00)	57.00 (0.00, 80.00)	−1.11	0.268
Ovarian hyperstimulation rate (%)	2.84 (4/141)	1.71 (11/645)	0.79	0.374

### The impact of the early LH surge on ART outcomes in subgroup cycles

During subgroup analysis (Table 2), patients with an early LH surge had a significantly higher spontaneous abortion rate than the control group in the IVF (25.93% vs 14.86, *P* = 0.049) and multiple embryo transfer (25.76 vs 13.50%, *P* = 0.014) subgroups. In addition, in the frozen embryo transfer cycle, patients exposed to an early LH surge appeared to have a higher preterm birth rate than those who did not (23.08 vs 12.50%, *P* = 0.093). Moreover, the live birth rate of patients with an early LH surge tended to be lower than those without the surge in IVF (37.76 vs 47.54%, *P* = 0.08) and multiple embryo transfer (40.37 vs 50.10%, *P* = 0.066) subgroups, respectively.

**Table 2:** Chi-squared comparison of ART outcomes between subgroup cycles. Data are presented as *n*/*n* (%).

Subgroups	Spontaneous abortion rate			Preterm birth rate			Live birth rate		
	LH surge (*n* = 84)	Control (*n* = 362)	*P*	LH surge (*n* = 59)	Control (*n* = 295)	*P*	LH surge (*n* = 141)	Control (*n* = 645)	*P*
Fresh/frozen									
Fresh	8/31 (25.81)	26/168 (15.48)	0.160	1/20 (5.00)	32/135 (23.70)	0.057	20/48 (41.67)	135/299 (45.15)	0.652
Frozen	12/53 (22.64)	30/194 (15.46)	0.218	9/39 (23.08)	20/160 (12.50)	0.093	39/93 (41.94)	160/346 (46.24)	0.459
Fertilization									
IVF	14/54 (25.93)	37/249 (14.86)	0.049*	28/37 (21.6)	35/203 (17.24)	0.523	37/98 (37.76)	203/427 (47.54)	0.080
ICSI	6/30 (20.00)	19/113 (16.81)	0.683	2/22 (9.09)	17/92 (18.48)	0.289	22/43 (51.16)	92/218 (42.2)	0.279
Transfer type									
Cleavage	14/61 (22.95)	35/236 (14.83)	0.128	6/43 (13.95)	36/192 (18.75)	0.458	43/101 (42.57)	192/429 (44.76)	0.691
Blastocyst	6/23 (26.09)	21/126 (16.67)	0.281	4/16 (25.00)	16/103 (15.53)	0.346	16/40 (40)	103/216 (47.69)	0.371
Embryo quality									
Top	14/60 (23.33)	43/283 (15.19)	0.124	9/41 (21.95)	41/230 (17.83)	0.530	41/94 (43.62)	230/469 (49.04)	0.337
Non-top	6/24 (25)	13/79 (16.46)	0.345	1/18 (5.56)	11/65 (16.92)	0.225	18/47 (38.3)	65/176 (36.93)	0.863
ET number									
Single	3/18 (16.67)	17/73 (23.29)	0.544	0/15 (0.00)	7/54 (12.96)	0.141	15/32 (46.88)	54/164 (32.93)	0.131
Multiple	17/66 (25.76)	39/289 (13.50)	0.014*	10/44 (22.73)	45/241 (18.67)	0.531	44/109 (40.37)	241/481 (50.1)	0.066

*P* represented the *P*-value of the *X*^2^ test.

* *P* < 0.05.

IVF, *in vitro* fertilization; ICSI, intracytoplasmic sperm injection; ET, embryo transfer.

Because both belong to univariate analysis, the chi-squared test (Table 2) and univariate logistic regression (Table 3) produced similar results when comparing ART outcomes. According to the univariate regression, the potential factors influencing pregnancy outcomes were adjusted in the multivariate logistic regression (see the note of Table 3). After controlling for covariates, the spontaneous abortion rate of the patients with an early LH surge increased significantly in the IVF and multiple embryo transfer subgroups (adjusted OR = 2.77, 95% CI = 1.30–5.92, *P* = 0.008 for the IVF subgroup, and adjusted OR = 2.42, 95% CI = 1.24–4.70, *P* = 0.009 for multiple embryo transfer subgroup). As a result, the live birth rate in the IVF (OR = 0.60, 95% CI = 0.38–0.96, *P* = 0.033) and multiple embryo transfer (OR = 0.64, 95% CI = 0.42–0.98, *P* = 0.040) subgroups decreased significantly (Table 3). In the frozen embryo transfer cycle subgroup, patients who experienced an early LH surge had a higher preterm birth rather than those who did not (OR = 2.92, 95% CI = 1.11–7.69, *P* = 0.031).

**Table 3:** Regression analysis of ART outcomes between subgroup cycles.

Outcome/subgroups	OR (95% CI)	*P*_u_	OR (95% CI)	*P*_m_
Spontaneous abortion rate				
Groups				
Fertilization				
IVF	2.01 (0.99–4.05)	0.052	2.77 (1.30–5.92)	0.008*
ICSI	1.24 (0.45–3.44)	0.683	1.08 (0.38–3.07)	0.887
ET number				
Single	0.66 (0.17–2.55)	0.546	0.96 (0.21–4.47)	0.955
Multiple	2.25 (1.18–4.32)	0.014*	2.42 (1.24–4.7)	0.009*
Preterm birth rate				
Groups				
Fresh/frozen				
Fresh	0.17 (0.02–1.32)	0.090	1.06 (0.05–23.29)	0.973
Frozen	2.10 (0.87–5.06)	0.098	2.92 (1.11–7.69)	0.031*
Live birth rate				
Groups				
Fertilization				
IVF	0.67 (0.43–1.05)	0.081	0.60 (0.38–0.96)	0.033*
ICSI	1.43 (0.74–2.76)	0.28	0.34 (0.09–1.33)	0.119
ET number				
Single	1.80 (0.83–3.87)	0.134	1.66 (0.77–3.61)	0.200
Multiple	0.67 (0.44–1.03)	0.067	0.64 (0.42–0.98)	0.040*

*P*_u_ and *P*_m_ represented the *P*-value of univariate and multivariate logistic regression, relatively, with * indicating *P* < 0.05. The following were the adjusted covariates:

Spontaneous abortion rate: i) basal LH, fasting glucose, good embryo numbers, and single/multiple embryo transfer cycles were adjusted in the IVF subgroup; ii) blastocyst number was adjusted in the ICSI subgroup; iii) age and COS day 5 progesterone level were adjusted in the single embryo subgroup; and iv) fasting glucose was adjusted in the multiple embryo subgroup.

Preterm birth rate: i) fasting glucose, single/multiple embryo transfer cycles, transfer embryo type, and LH levels on COS day 5/6 were adjusted in the fresh embryo transfer subgroup and ii) FSH levels on hCG day, transfer embryo type, and single/multiple embryo transfer cycles were adjusted in the frozen embryo transfer subgroup.

Live birth rate: i) basal LH level and good embryo numbers were adjusted in the IVF subgroup; ii) basal LH level, COS day 5 LH level, and single/multiple embryo transfer cycles were adjusted in the ICSI subgroup; iii) basal FSH level was adjusted in the single embryo transfer subgroup; and iv) good embryo numbers were adjusted in the multiple embryo transfer subgroup.

IVF, *in vitro* fertilization; ICSI, intracytoplasmic sperm injection; ET, embryo transfer.

### Construction of predictive models using the training set

**Figure 2:**
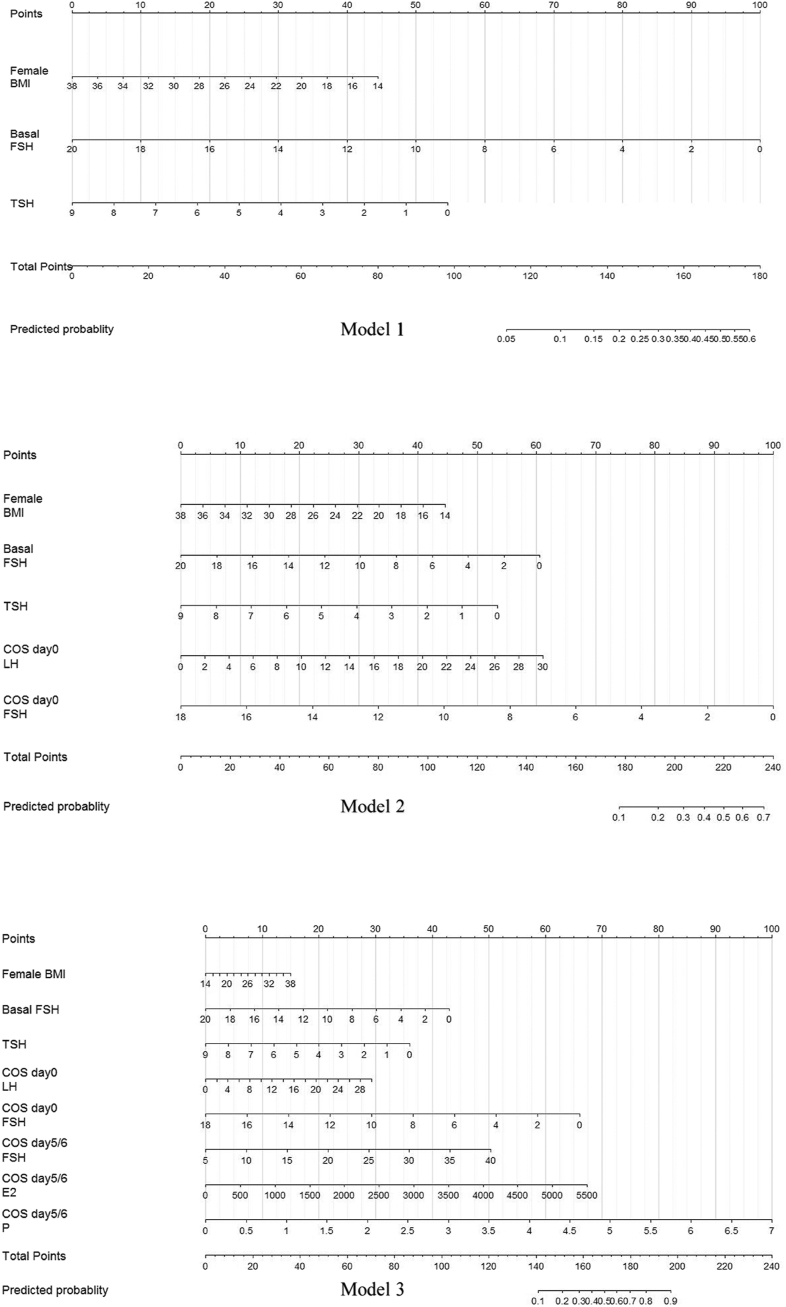
Nomograms for quantifying the probability of the early LH surge.

We generated the predictive models using a training set of 564 patients recruited between January 1, 2014, and April 1, 2020. The risk factors that contributed to an early LH surge were identified using multivariate logistic regression. Based on the risk factors, we established three models to predict the early LH surge before COS, on COS day 0, and on COS day 5 (Table 4). Before COS in model 1, the independent prognostic factors entered into the algorithm were BMI (OR = 0.91, 95% CI = 0.84–0.98, *P* = 0.008), basal FSH (OR = 0.77, 95% CI = 0.67–0.88, *P* < 0.001), and basal TSH (OR = 0.73, 95% CI = 0.56–0.94, *P* = 0.017): logit *P* = 2.93 − 0.10 * (BMI (kg/m^2^)) − 0.26* (basal FSH (IU/L)) − 0.32 * (TSH (mU/L)). Based on model 1, on COS day 0, LH and COS day 0 FSH were added to the equation of model 2 to estimate the probability of an early LH surge: logit *P* = 3.39 − 0.10 * (BMI (kg/m^2^)) − 0.16 * (basal FSH (IU/L)) − 0.31 * (TSH (mU/L)) + 0.11 (COS day 0 LH (IU/L)) − 0.29 (COS day 0 FSH (IU/L)). On COS day 5, FSH, E_2_, and progesterone were also incorporated into the algorithm: logit *P* = −3.10 + 0.0486 * (BMI (kg/m^2^)) − 0.17 * (basal FSH (IU/L)) − 0.31 * (TSH (mU/L)) + 0.08 (COS day 0 LH (IU/L)) − 0.29 (COS day 0 FSH (IU/L)) + 0.11 (COS day 5/6 FSH (IU/L)) + 0.001 (COS day 5/6 E_2_ (ng/mL)) + 1.12 (COS day 0 progesterone (IU/L)). Besides, the nomograms were created to quantifythe probability of an early LH surge on three occasions (Fig. 2). In the nomograms, each clinical parameter corresponded to a specific point, which was then summed up to yield a total point.The probability of an early LH rise would be calculated from the total points based on the above equations.

**Table 4:** Various models for forecasting the early LH surge.

Variables	Model 1: AUC = 0.672		Model 2: AUC = 0.713		Model 3: AUC = 0.881	
	OR (95%CI)	*P*	OR (95%CI)	*P*	OR (95%CI)	*P*
Intercept	-	0.004	-	0.005	-	0.041
BMI	0.91 (0.84–0.98)	0.008	0.91 (0.84–0.98)	0.014	1.05 (0.96–1.15)	0.291
Basal levels
FSH	0.77 (0.67–0.88)	<0.001	0.85 (0.73–1.00)	0.053	0.84 (0.69–1.03)	0.091
TSH	0.73 (0.56–0.94)	0.017	0.73 (0.56–0.97)	0.028	0.73 (0.53–1.01)	0.057
COS day 0
LH	-	-	1.11 (1.04–1.19)	0.004	1.08 (0.98–1.19)	0.123
FSH	-	-	0.75 (0.62–0.91)	0.003	0.75 (0.61–0.92)	0.007
COS day 5/6
FSH	-	-	-	-	1.12 (1.04–1.20)	0.003
E_2_	-	-	-	-	1.00 (1.00–1.00)	<0.001
Progesterone	-	-	-	-	3.06 (1.70–5.52)	<0.001

Model 1: BMI, basal FSH, and basal TSH levels before ART; Model 2: Model 1, LH, and FSH levels on COS day 0; Model 3: Model 2, FSH, E_2_, and progesterone levels on COS day 5/6. BMI, body mass index; COS, controlled ovarian stimulation.

### Predictive model validation on the time validation set

**Figure 3:**
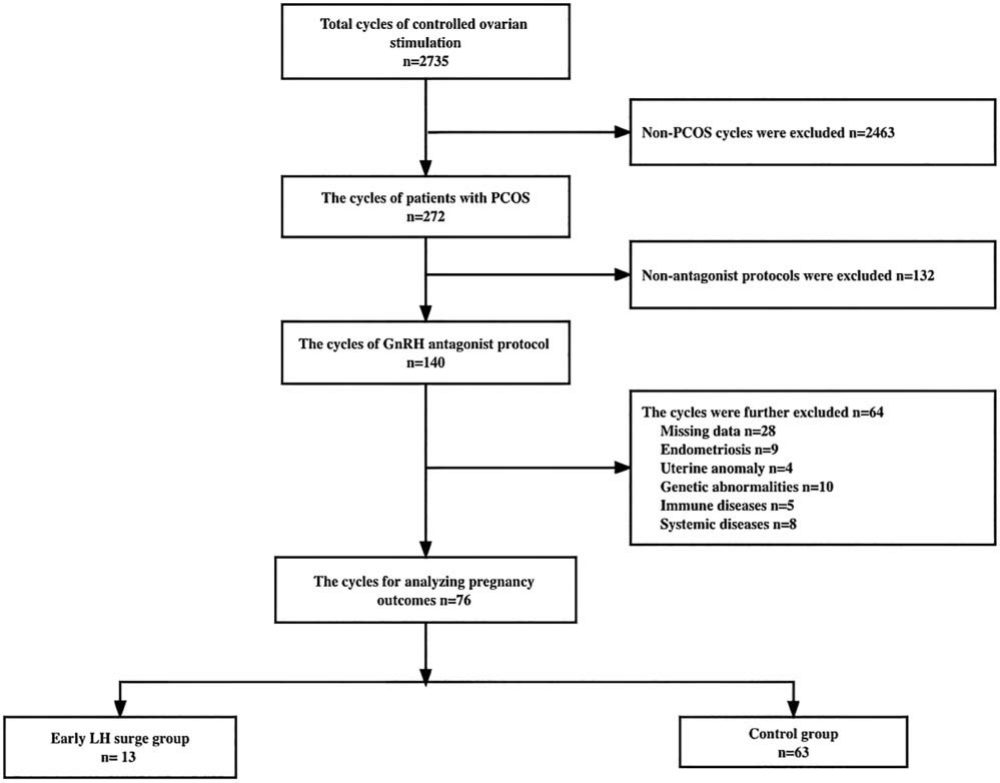
Flowchart for recruitment of patients with PCOS with and without an early LH surge in the time validation set.

**Figure 4:**
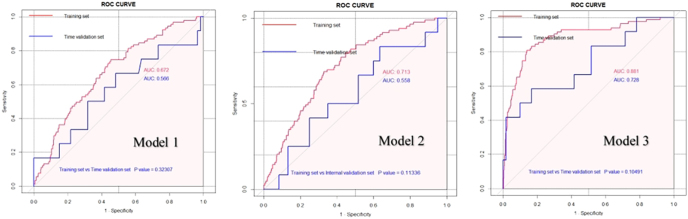
ROC curves of the training and time validation sets.

Furthermore, we performed time validation of predictive models (training set) on another cohort of 76 patients recruited at our center between January 1, 2021, and December 31, 2021 (Fig. 3). The area under the curve (AUC) of training and time validation sets was 0.672 vs 0.566 (before COS), 0.713 vs 0.554 (COS day 0), and 0.881 vs 0.728 (COS day 5), indicating that the predictive models had satisfactory goodness of fit (*P* = 0.323, 0.113, and 0.105, respectively) (Fig. 4). The calibration curves also showed good consistency between estimated probabilities and actual results. Moreover, the DCA schemes demonstrated a reasonable utility of those models in clinical decision-making (Supplemental Figs 1, 2, and 3, Supplementary materials).

### The risk cutoff values of the nomogram and a high-risk patient intervention pathway map

The cut-off values were determined using the maximum Youden index in ROC cure, representing the point that optimally balances sensitivity and specificity. The two-tailed *P* < 0.05 was considered a significant difference. The cut-off values represent the probability threshold points used by the model to distinguish between the positive and negative classes when making binary classification decisions. As shown in Supplemental Fig. 4, the cut-off value was 0.151 in model 2.

An intervention pathway map would be developed based on the predictive models. Fox example, if a patient reached the cut-off value on COS day 0 based on model 2, which indicates a high risk of premature LH surge, the clinician may consider starting an early use of GnRH antagonist on COS day 3/4 to prevent the early LH surge.

## Discussion

Reproductive clinicians usually ignore the early LH surge that occurs in patients with PCOS because the surge is transient and the patients have good ovarian reserve. Nonetheless, after stratifying the patients and adjusting for potential covariates, our study revealed that the early LH surge might have a detrimental impact on ART outcomes in specific subgroups of patients with PCOS (IVF, multiple embryo transfer, and frozen embryo transfer cycle). Therefore, we developed three models to identify high-risk patients, and after trading off the predictive timing and power, we propose using model 2 in clinical practice. Furthermore, we performed time validation of those models in a prospective cohort of patients with PCOS, which demonstrated consistent predictive value.

Referring to the previous reports, this study combined absolute and relative LH levels to define the early LH surge (Choi *et al.* 2013, Gao *et al.* 2021). The progesterone level was not included in the criteria because the early LH surge may not fully luteinize the granulosa cells after adding the GnRH antagonist. In our results, the average progesterone level on COS day 5/6 was below 2 ng/mL, which did not meet our center’s luteinizing standard. According to the characteristics, the patients with early LH surge had a better ovarian reserve and acquired more eggs. Their blastocyst quantity, however, was comparable to those without the surge, implying that the LH surge could impair the oocyte developmental competence. Interestingly, similar to the basal FSH level, the basal TSH level in these patients was significantly lower. TSH and FSH, both glycoprotein hormones released by the anterior pituitary gland, share extensive similarities in the β-subunit amino acid sequence, which might explain their similar pituitary response (Escudero *et al.* 2005, Núñez Miguel *et al.* 2008, Cahoreau *et al.* 2015). The elevated basal levels of LH and PRL suggested that those patients may have a hypothalamic–pituitary axis sensitive to the positive feedback of estrogen. During ovarian stimulation, their estradiol levels rose higher and faster, contributing to the early LH surge via positive feedback.

Only a few studies have looked into the relationship between a transient LH surge during the early stimulation phase and ART outcomes. Zhang *et al.* (2019) reported that a transient LH surge had an adverse impact on pregnancy outcomes, but this study investigated the general population, not focused on the patients with PCOS (Szkudlinski *et al.* 2002). After stratifying the cycles based on key clinical cofounders, the spontaneous abortion rates of patients with an early LH surge increased significantly in the IVF and multiple embryo transfer subgroups. According to univariate analyses such as the chi-squared test and univariate logistic regression, in which many confounders were not adjusted, the preterm birth and live birth rates tended to increase in the patients with an early LH surge. However, after adjusting for potential cofounders identified in the univariate logistic regression, both variables showed significant differences. Surprisingly, LH and progesterone levels on hCG day did not play a significant role in compromising ART outcomes, which could be explained by the fact that adding a GnRH antagonist may profoundly suppress LH surge and render the LH and progesterone levels on hCG day less relevant to pregnancy outcomes.

According to the previous studies, a premature LH surge during COS could disrupt oocyte maturation and jeopardize embryo competence (Qiao & Feng 2010, Dovey *et al.* 2011, Zhang *et al.* 2019). Premature LH rise, in particular, will disrupt the meiotic process in oocytes and the extrusion of the first polar body, which might increase the formation of aneuploidy embryos, the primary cause of spontaneous abortion (Tarlatzis & Grimbizis 1997, Jonard 2004, Kristensen *et al.* 2022). Furthermore, the miscarriage rate in the multiple embryo transfer subgroup was higher than in the single embryo transfer subgroup. The reason for this could be that the multiple embryo transfer strategy amplifies the negative impact of an early LH surge on oocyte quality. Notably, miscarriage and live birth outcomes worsen in the IVF subgroup but not in the ICSI subgroup, possibly due to male factors interfering with those outcomes in the ICSI cycle. In addition, the preterm birth rate increased during the frozen cycle, indicating embryos in the case group might be vulnerable to the frozen protocol.

Moreover, this study established three predictive models for identifying high-risk patients on various occasions. Model 1 can forecast the risk of early LH rise at the earliest moment, but it has the lowest predictive power of the three models. Model 3 has the highest predictive power as more significant variables are introduced to the algorithm, but it would be too urgent for clinicians to adjust the stimulating protocol on COS day 5/6. After balancing the predictive timing and power, we recommend utilizing model 2 in clinical practice. In addition, nomograms were created to quantify the probability of an early LH surge using total points transformed from clinical parameters. Finally, a prospective cohort of patients was enrolled for the time validation, demonstrating the models’ potential clinical applicability.

Our findings were consistent with the prior research, which reported a lower ongoing pregnancy rate in the flexible protocol than in the fixed protocol (Dumesic *et al.* 2008). As shown by our data, the PCOS patients with basal LH ≧ 10 IU/L who were co-administrated with GnRH antagonist and gonadotropin on the initial day had similar clinical pregnancy rates but significantly lower miscarriage rates than those who were administrated with GnRH antagonist on COS day 5/6 (Franks *et al.* 2008). Another study demonstrated that a profound suppression of LH on day eight during ovarian stimulation was associated with a higher ongoing pregnancy rate, implying the significance of controlling LH fluctuation (Kolibianakis *et al.* 2003). Moreover, some clinicians administered the progestin-primed ovarian stimulation (PPOS) protocol in PCOS patients, effectively preventing the premature LH surge and improving pregnancy outcomes. During the PPOS protocol, the progestins are usually added on the initial stimulation day (Kolibianakis *et al.* 2004, Huang *et al.* 2020, Chen *et al.* 2022). Taken together, if a PCOS patient is at high risk of premature LH rise, as predicted by the model on the first day of COS, it is preferable to administer a GnRH antagonist prior to COS day 5/6. However, more randomized clinical trials are still needed to confirm this strategy.

This study reveals that an early LH surge may compromise the ART outcomes in certain subsets of patients with PCOS. Besides, three predictive models have been developed to help clinicians identify high-risk patients and take preventive measures to improve ART outcomes. The first strength of this study is that the patients were stratified into various subgroups based on critical clinical factors. In addition, the potential cofounding factors were adjusted during statistical analysis. Third, we established different predictive models to assist clinicians in identifying high-risk patients and taking preventive measures. The limitations of this study include insufficient statistical power of small-sample subgroup analysis and failure to exclude the embryo selection bias. Moreover, subgroup analyses may increase the risk of type 1 error due to multiple comparisons. Besides that, we must acknowledge that some lacking confounding factors may decrease the strength of the conclusions due to data limitations.

In summary, an early LH surge during ovarian stimulation may jeopardize the ART outcomes in specific subgroups of patients with PCOS, including *in vitro* fertilization, multiple embryo transfer, and frozen embryo transfer cycles. Furthermore, we develop predictive models to help clinicians identify high-risk patients on different occasions. By trading off the predictive timing and power between three models, we recommend utilizing model 2 in clinical practice because clinicians would have more flexibility to adjust the timing of adding the GnRH antagonist. Moreover, the efficacy of those models has been confirmed by a prospective cohort of patients in the time validation set. A multi-center study with more patients in the future is still needed to validate the clinical applicability of those models.

## Supplementary materials



## Declaration of interest

The authors declare that there is no conflict of interest that could be perceived as prejudicing the impartiality of the work reported.

## Funding

We received the support from the National Key Research and Development Program of Chinahttps://doi.org/10.13039/501100012166 (Grant No. 2019YFA0801403) to collect the data and the support from the National Natural Science Funding (CN) (Grants Nos. 81671523, 81971332, and 82171642), the Natural Science Funding of Guangdong Province (CN) (Grants Nos. 4210015092 and 2017A030313895), the Guangzhou Science and Technology Plan (Grants No. 202201010782), and the Funding of Yat-sen Scholarship for Young Scientist to perform statistical analysis.

## Author contribution statement

XJ, HX, and TL collected patient data and conducted statistical analysis; HL and WQ followed up patients’ outcomes; YY performed the hormone measurement; and QZ and HC recruited the patients; YL, LW, and QQ designed the study and wrote the manuscript.

## Ethics approval

This study was approved by the Institutional Review Board (IRB) before initiating the project (IRB No. SYSKY-2024-1080-01).
