# Supplementary figures and images for: An early LH surge may jeopardize ART outcomes of patients with PCOS and its predictive models
Source: Reprod Fertil. 2026 Mar 4;7(1):RAF250160. doi: 10.1530/RAF-25-0160 (PMC12974764; doi:10.1530/RAF-25-0160)

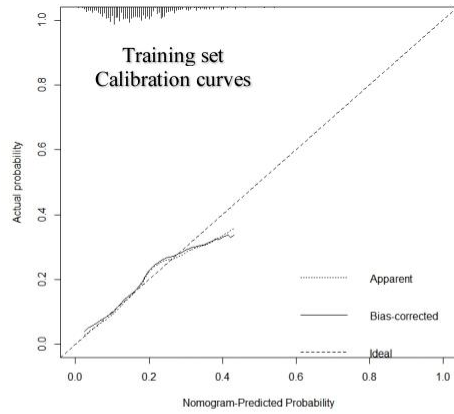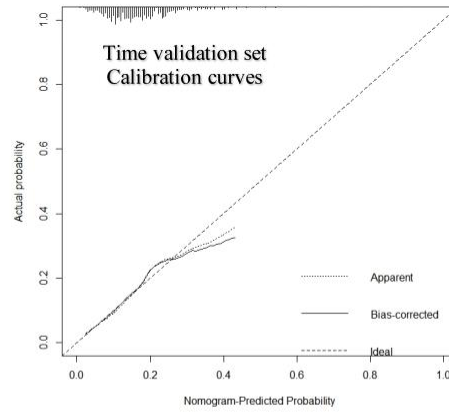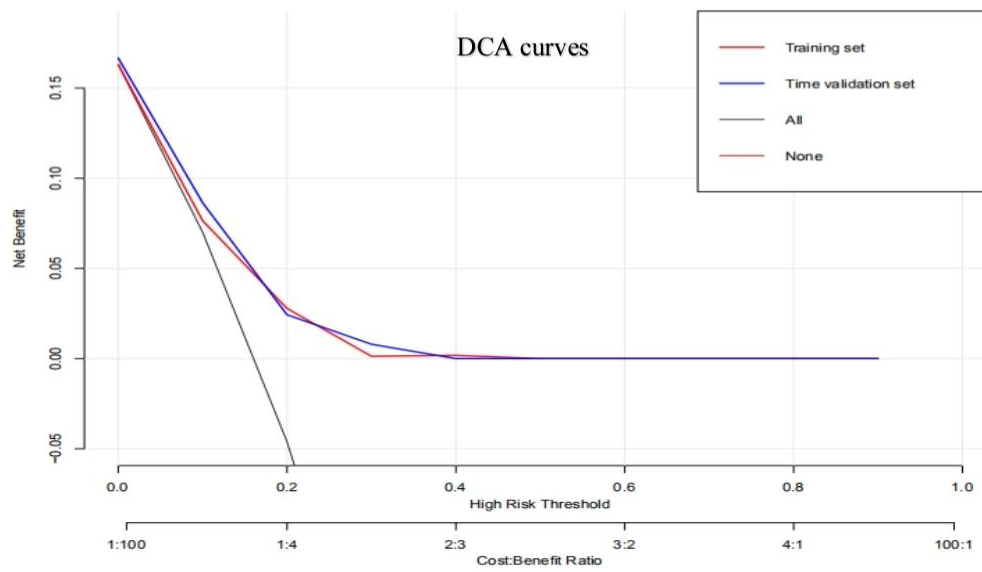

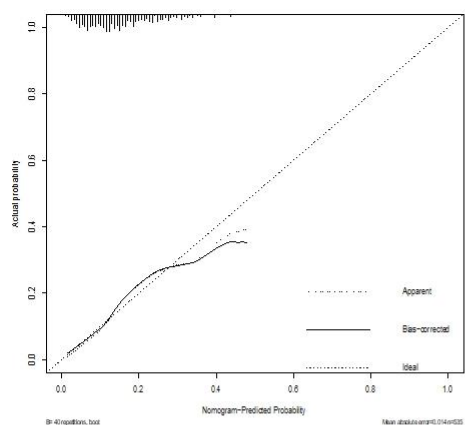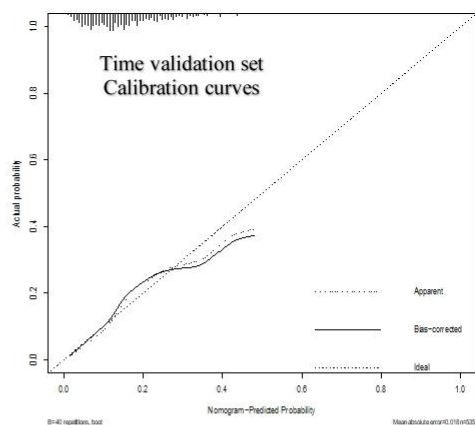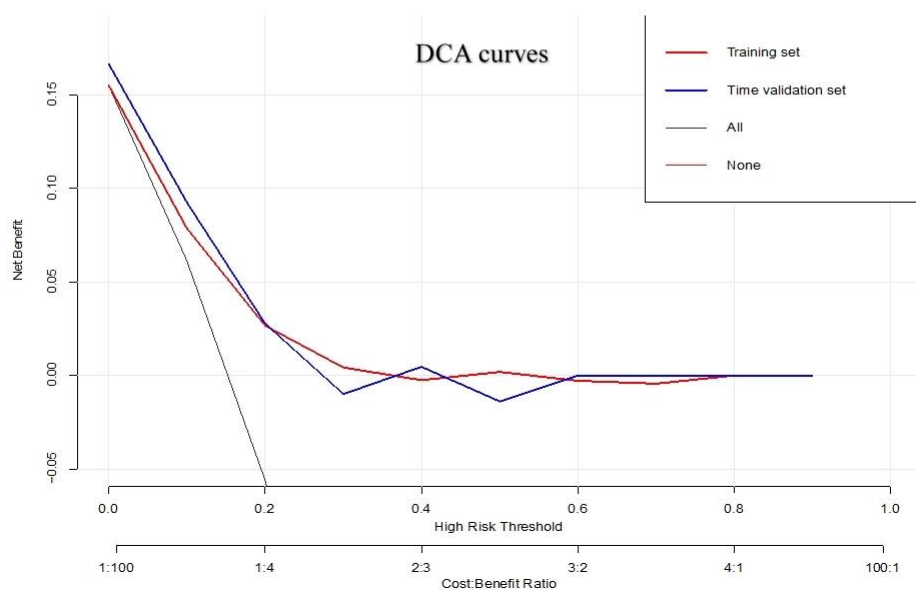

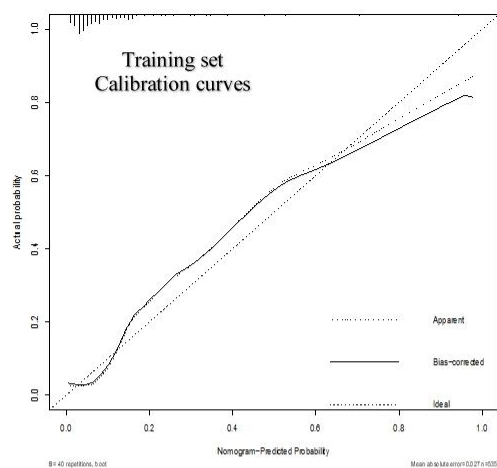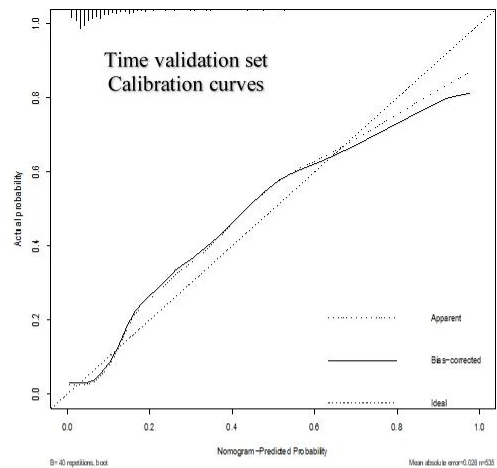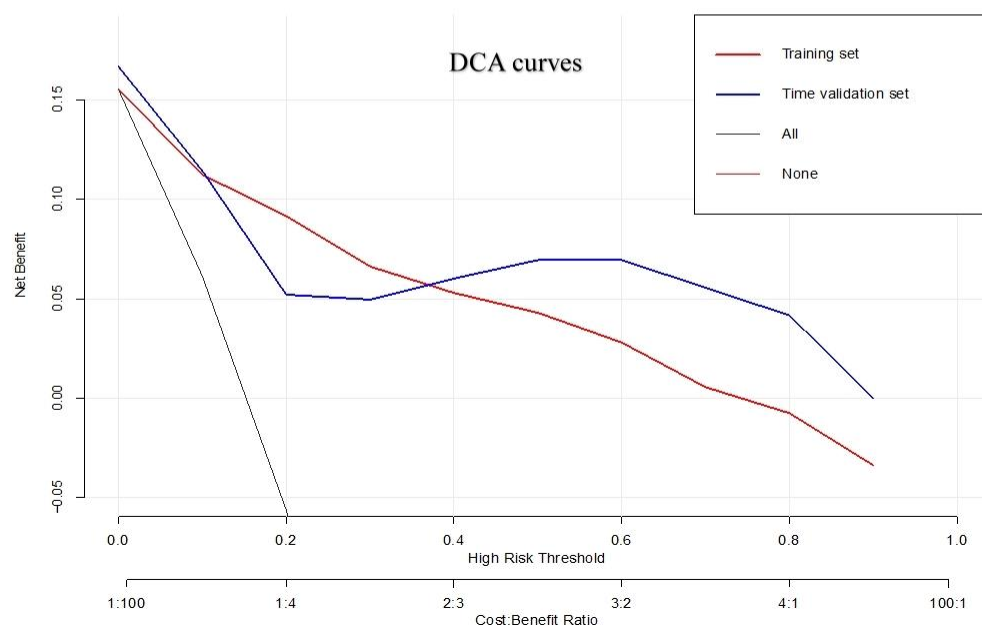

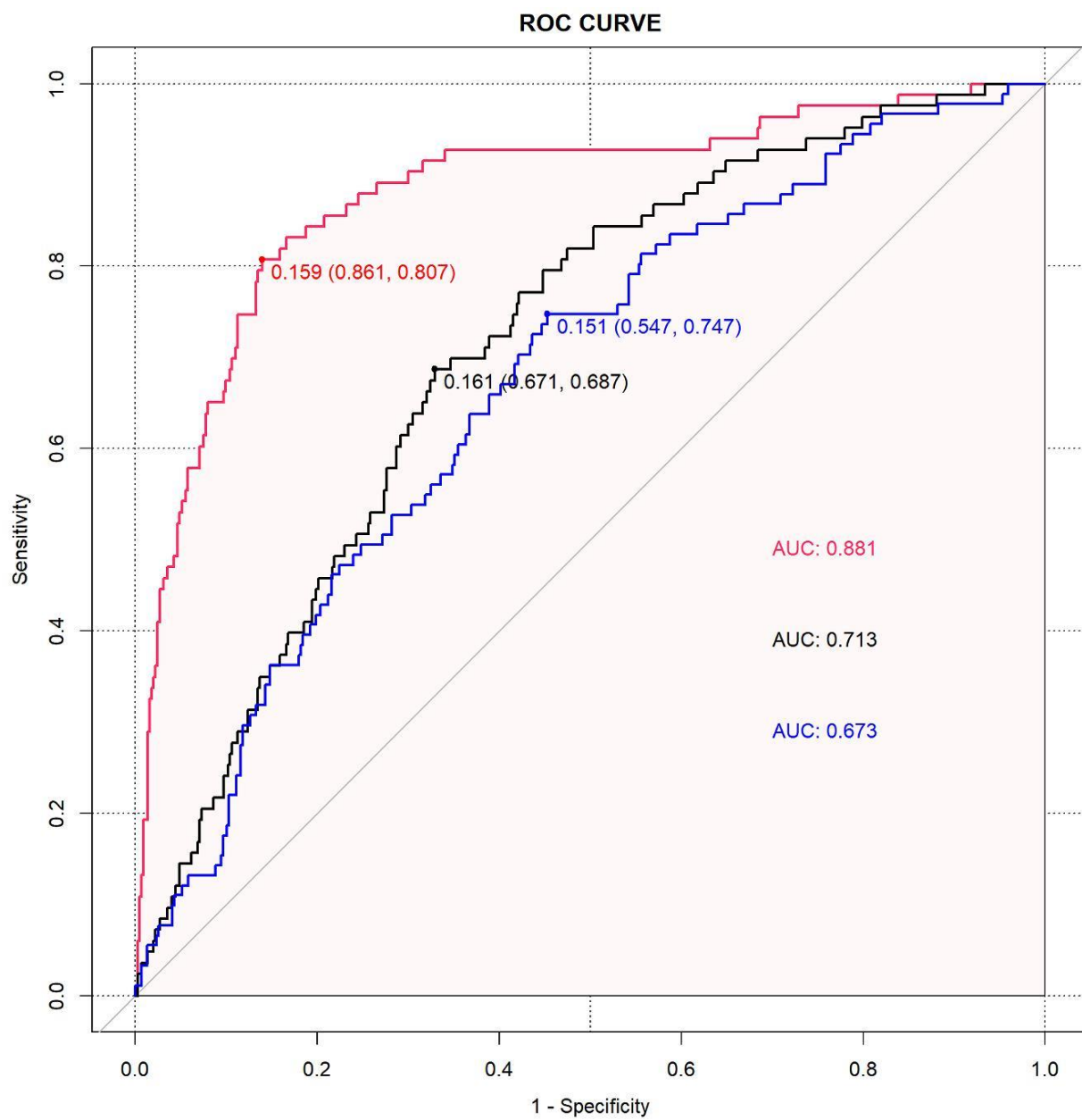

Supplement: Supplementary file 1 [file supplementary_materials.pdf]
